# Anti-Inflammatory Effects of *Lasia spinosa* Leaf Extract in Lipopolysaccharide-Induced RAW 264.7 Macrophages

**DOI:** 10.3390/ijms21103439

**Published:** 2020-05-13

**Authors:** Thanh Q. C. Nguyen, Tran Duy Binh, Tuan L. A. Pham, Yen D. H. Nguyen, Dai Thi Xuan Trang, Trong Tuan Nguyen, Kenji Kanaori, Kaeko Kamei

**Affiliations:** 1 Department of Functional Chemistry, Kyoto Institute of Technology, Kyoto 606-8585, Japan; nqcthanh@ctu.edu.vn (T.Q.C.N.); tdbinh22@gmail.com (T.D.B.); phamleanhtuan.2807@gmail.com (T.L.A.P.); ndhyen94@gmail.com (Y.D.H.N.); kanaori@kit.ac.jp (K.K.); 2 Department of Chemistry, College of Natural Sciences, Can Tho University, Cantho City 94000, Vietnam; trongtuan@ctu.edu.vn; 3 Department of Biology, College of Natural Sciences, Can Tho University, Cantho City 94000, Vietnam; dtxtrang@ctu.edu.vn

**Keywords:** *Lasia spinosa*, anti-inflammatory, macrophages, inflammatory cytokines, anti-oxidant

## Abstract

*Lasia spinosa* (L.) Thwaites was used as a traditional medicine to treat many inflammatory diseases for centuries. However, its effects on the inflammatory response are not yet characterized. In this study, we investigated the anti-inflammatory activities of *L. spinosa* leaf extract in lipopolysaccharide (LPS)-induced RAW 264.7 macrophages. We found that ethanol extracts of *L. spinosa* leaves showed anti-oxidant activity due to the presence of high levels of polyphenolic compounds. Treatment with the leaf extract significantly repressed the production of inflammatory mediators such as nitric oxide and reactive oxygen species and the expression of pro-inflammatory cytokines in the LPS-stimulated RAW 264.7 cells. Moreover, *L. spinosa* leaf extract treatment prevented activation of the nuclear factor-kappa B pathway by inhibiting nuclear factor of kappa light polypeptide gene enhancer in B-cells inhibitor, alpha (IκBα) degradation. Furthermore, the mitogen-activated kinase and phosphoinositide-3-kinase/protein kinase B (PI3K/Akt) pathways were suppressed upon treatment with the leaf extract. In addition to suppressing inflammatory factors, the extract also activated the nuclear factor erythroid 2-related factor 2/heme-oxygenase-1 pathway. We propose that *L. spinosa* leaf extract has the potential as an effective therapeutic agent for alleviating oxidative stress and excessive inflammation.

## 1. Introduction

Inflammation is a mechanism used to defend against various infections and injuries; it is a well-appreciated aspect of complex biological responses that maintain homeostasis in the human body [1]. However, chronic inflammatory processes are implicated in the pathogenesis of common inflammation-associated diseases such as rheumatoid arthritis, chronic hepatitis, pulmonary fibrosis, and cancer [2]. Notably, innate immune cells (particularly macrophages) initiate an inflammatory response via the overwhelming production of pro-inflammatory mediators, such as nitric oxide (NO) and prostaglandin E_2_ (PGE_2_), and inflammatory cytokines such as tumor necrosis factor-α (TNF-α), interleukin-1β (IL-1β), and interleukin-6 (IL-6) [3]. As such, levels of pro-inflammatory mediators and cytokines are reflective of the degree of inflammation, and are used to evaluate the effect of pharmacological agents on inflammatory processes. In particular, lipopolysaccharide (LPS), a bacterial endotoxin, can induce inflammation signaling pathways via Toll-like receptor 4 (TLR-4), which stimulates the recruitment of cytoplasmic MyD88 and Toll like receptor-domain-containing adapter-inducing interferon-β (TRIF) adaptor proteins to increase the secretion of pro-inflammatory mediators and cytokines [4,5]. LPS initiates a signaling cascade and subsequent activation of the nuclear factor-kappa B (NF-κB) pathway, the mitogen-activated protein kinase (MAPKs: extracellular signal-regulated kinases 1/2 (ERK 1/2), c-Jun N-terminal kinase (JNK), and p38 MAPK) pathways, and the phosphatidylinositol (PI) 3-kinase (PI3K)/protein kinase B (Akt) signaling pathway [6]. Inflammation is related to oxidative stress, which elevates the levels of intracellular reactive oxygen species (ROS) and regulates the production of antioxidant enzymes [7]. The induction of phase II detoxifying enzymes, including heme oxygenase-1 (HO-1), is controlled by translocation of the nuclear factor-erythroid-2-related factor (Nrf2) in the Nrf2/HO-1 signaling pathway, which is a master cellular sensor that protects cells against inflammation and oxidative stress [8].

Nonsteroidal anti-inflammatory drugs (NSAIDs) are used to treat many inflammatory diseases. These drugs attack cyclooxygenase-2 (COX-2) and inhibit the synthesis of prostaglandins, thereby causing undesirable side effects such as toxicity and myocardial infarction [9]. These issues necessitate the development of novel agents to treat acute inflammation and chronic inflammatory diseases in a more effective and less toxic manner [10]. Because of the side effects associated with NSAIDs, natural compounds are used as dietary supplements and herbal treatments to reduce pain and inflammation. Many of these natural compounds and herbs inhibit inflammatory pathways to a similar degree as NSAIDs [11].

*Lasia spinosa* (L.) Thwaites (LS) is used in traditional Vietnamese medicine to alleviate the symptoms of many inflammatory diseases. Furthermore, the phytochemicals in LS include alkaloids, polyphenols, and flavonoids, which have anti-oxidant, anti-bacterial, anti-hyperglycemic, and anti-cancer properties [12,13,14]. A recent study showed that *L. spinosa* leaf extract possesses a considerable level of efficacy against *Trichinella spiralis* infections in mice [15]. In this study, we examined the anti-inflammatory properties of ethanol extracts from LS leaves in LPS-stimulated RAW 264.7 macrophages. We found that LS leaf ethanol extracts inhibited activation of the TLR-4 pathway and promoted the Nrf2/HO-1 pathway. Thus, we demonstrate that LS could be used as a potential anti-inflammatory agent.

## 2. Results

### 2.1. Antioxidant Activity of LS Leaf Ethanol Extracts

**Figure 1:**
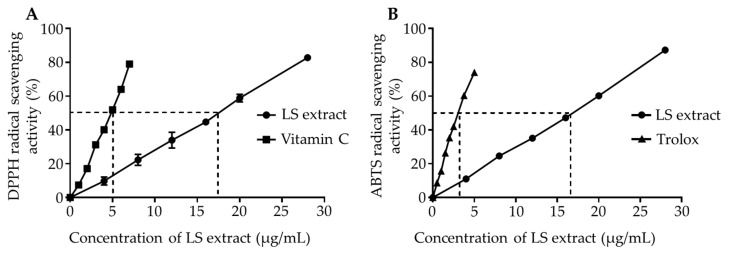
Radical-scavenging activity of the *Lasia spinosa* (LS) leaf ethanol extract. Varying concentrations of LS leaf extract were mixed with 2,2-diphenyl-1-picrylhydrazyl (DPPH) (**A**) or 2,2-azino-bis(3-ethylbenzothiazoline-6-sulfonic acid) diammonium salt (ABTS) (**B**). Vitamin C and Trolox were used as positive controls in (**A**) and (**B**), respectively. After incubation, the absorbance at 517 nm in the DPPH assay or 734 nm in the ABTS assay was measured, and radical scavenging activity was calculated. The dotted line indicates the concentration of LS leaf extract or control substance which is required for 50% radical scavenging (SC_50_). Data are expressed as means ± SD. Significant differences between the LS leaf extract and vitamin C or Trolox controls (*n* = 3) were evaluated by *t*-test.

To test the antioxidant activity of LS leaf ethanol extracts, radical scavenging ability was indirectly evaluated by DPPH (2,2-diphenyl-1-picrylhydrazyl) and ABTS (2,2-azino-bis(3-ethylbenzothiazoline-6-sulfonic acid) diammonium salt) assays. The free radical-scavenging activities increased linearly with increasing concentration of LS leaf extract in both assays (Figure 1). The concentrations of LS leaf extract required to scavenge 50% of the free radicals (SC_50_) in the DPPH and ABTS assays were 17.25 μg/mL (equivalent to 5.38 μg/mL vitamin C) and 16.47 μg/mL (equivalent to 3.17 μg/mL Trolox), respectively (Figure 1).

Additionally, the total amount of polyphenols expressed as gallic acid equivalent (GAE) was 158 ± 0.56 mg GAE/g of extract (20.08 ± 0.07 mg GAE/g dried leaf). These results demonstrated that the LS leaf ethanol extract could effectively scavenge free radicals.

### 2.2. Effect of LS Leaf Extract on RAW 264.7 Cells Viability

**Figure 2:**
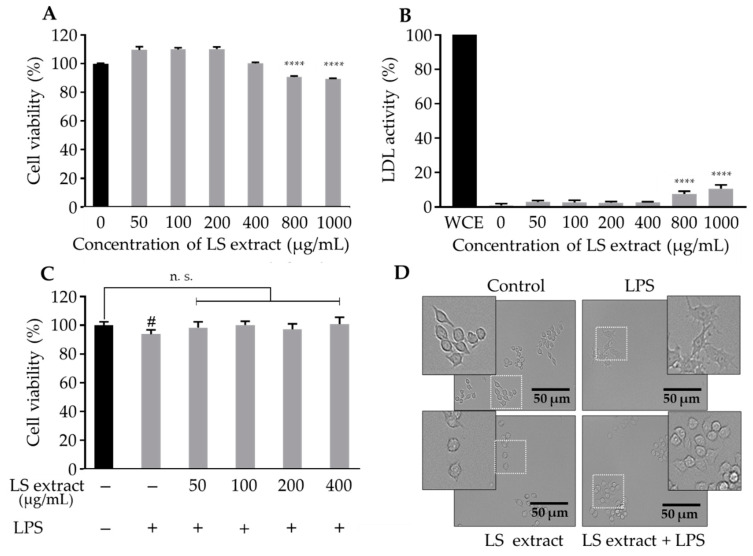
Effects of LS leaf extract on cell viability and morphology of RAW 264.7 cells. RAW 264.7 macrophages were cultured with LS leaf extract for 24 h, and then cell viability based on intracellular dehydrogenase activity (**A**) and cytotoxicity based on lactate dehydrogenase (LDH) activity released in culture medium (**B**) were measured. Whole-cell extract (WCE) was prepared as a positive control representing 100% activity in (**B**). After pretreatment with LS extract for 24 h, RAW 264.7 cells were stimulated by LPS (1 µg/mL) for 18 h, and then cell viability was measured (**C**). RAW 264.7 cells were treated with medium only (control), LPS, or LS extract (400 µg/mL) for 24 h. The LS extract + LPS group was treated with LPS (1 µg/mL) for 18 h. Cell morphology was observed under a phase-contrast microscope (**D**). Magnification of the region framed with a dotted white line is shown in the frame with a black line. Values represent the mean ± SD (*n* = 6). Statistical significance was calculated by *t*-test and one-way ANOVA. Scale bar in (**D**), 50 µm; # *p* < 0.05; **** *p* < 0.0001 vs. cells treated with media only; n. s., not significant.

The cytotoxicity of LS leaf extract was examined using RAW 264.7 cells in order to find the optimal concentration (effective in providing anti-inflammatory with minimum toxicity). At an LS leaf extract concentration of 800 µg/mL, cell survival was reduced by ~10% (Figure 2A) and lactate dehydrogenase (LDH) activity was increased (Figure 2B), suggesting cell toxicity. No cytotoxicity was observed with a concentration of LS leaf extract (<400 µg/mL).

Since LPS is known to reduce cell viability, the cytotoxicity of a mixture of LS leaf extract and LPS was also examined. As shown in Figure 2C, cell survival was significantly reduced by LPS but not by the mixture of LPS and LS leaf extract (<400 µg/mL). The LPS-stimulated cells had an abnormal shape (spindle-shaped pseudopodia and spreading) (Figure 2D). This phenomenon is one of the characteristics of LPS-activated RAW 264.7 macrophages [16]. Consistent with the above result, RAW 264.7 cells did not show the abnormal shape by treatment with a mixture of LPS and LS leaf extract at 400 µg/mL (Figure 2D). Taken together, these data indicate that the viability of RAW 264.7 cells is not affected by the LS leaf extract (<400 µg/mL), and the LS leaf extract protects the cell from LPS-induced toxicity.

### 2.3. LS Leaf Extract Inhibits the Production of NO, ROS, and TNF-α in LPS-Stimulated RAW 264.7 Cells

**Figure 3:**
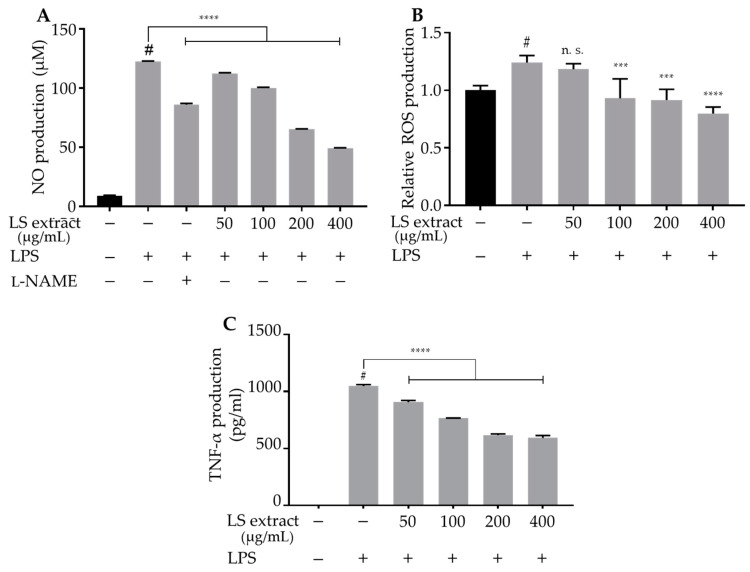
Effects of LS leaf extract on the production of nitric oxide (NO), reactive oxygen species (ROS), and tumor necrosis factor-alpha (TNF-α) in lipopolysaccharide (LPS)-stimulated RAW 264.7 cells. Cells were pretreated with LS leaf extract for 24 h. After washing with phosphate-buffered saline (PBS), cells were stimulated with 1 µg/mL LPS for 18 h. The level of NO in the medium was quantified by Griess reagent (**A**), and cellular ROS concentration was analyzed (**B**). *N*-Nitro-L-arginine methyl ester (L-NAME; 100 µM) was used as a control in the NO assay. The levels of TNF-α in RAW 264.7 cells were analyzed by an ELISA (**C**). Values represent the mean ± SD (*n* = 3). Statistical significance was calculated by *t*-test and one-way ANOVA. # *p* < 0.05 vs. cells treated with media only; n. s., not significant; *** *p* < 0.001 and **** *p* < 0.0001 vs. cells treated with LPS only.

NO, a signaling molecule produced by inducible nitric oxide synthase (iNOS), plays an essential role in the inflammatory response and is produced at higher concentrations in response to inflammation or injury [17,18]. To investigate the anti-inflammatory effect of LS leaf extract in LPS-stimulated RAW 264.7 cells, the amount of NO in culture medium was quantified. *N*-Nitro-L-arginine methyl ester (L-NAME) (100 µM) was used as a positive control. NO production was significantly increased by stimulation with LPS; however, pretreatment with LS leaf extract (50–400 µg/mL) significantly inhibited NO production in a dose-dependent manner (Figure 3A).

ROS are signaling molecules that play an essential role in the progression of inflammatory disorders [19]. Thus, we evaluated the effect of pretreatment with LS leaf extract on intracellular ROS production in LPS-stimulated RAW 264.7 cells. Figure 3B demonstrates that ROS production was upregulated by LPS-stimulation and significantly reduced by pretreatment with LS leaf extract (except for the 50 µg/mL dose). These results indicate that LS leaf extract not only suppresses NO production but also inhibits intracellular ROS production in LPS-stimulated RAW 264.7 cells.

Since TNF-α is a strong pro-inflammatory cytokine that plays a crucial role in the immune system during inflammation [20], its expression in RAW 264.7 cells was analyzed using an enzyme-linked immunosorbent assay (ELISA). As illustrated in Figure 3C, LPS-stimulated RAW 264.7 cells increased TNF-α levels, while pretreatment with LS leaf extract (50–400 μg/mL) significantly suppressed TNF-α expression. Taken together, these data suggest that the LS leaf extract suppresses the production of inflammatory mediators in LPS-stimulated RAW 264.7 cells.

### 2.4. LS Leaf Extract Inhibits the Expression of Inflammatory Genes in LPS-Stimulated RAW 264.7 Cells

**Figure 4:**
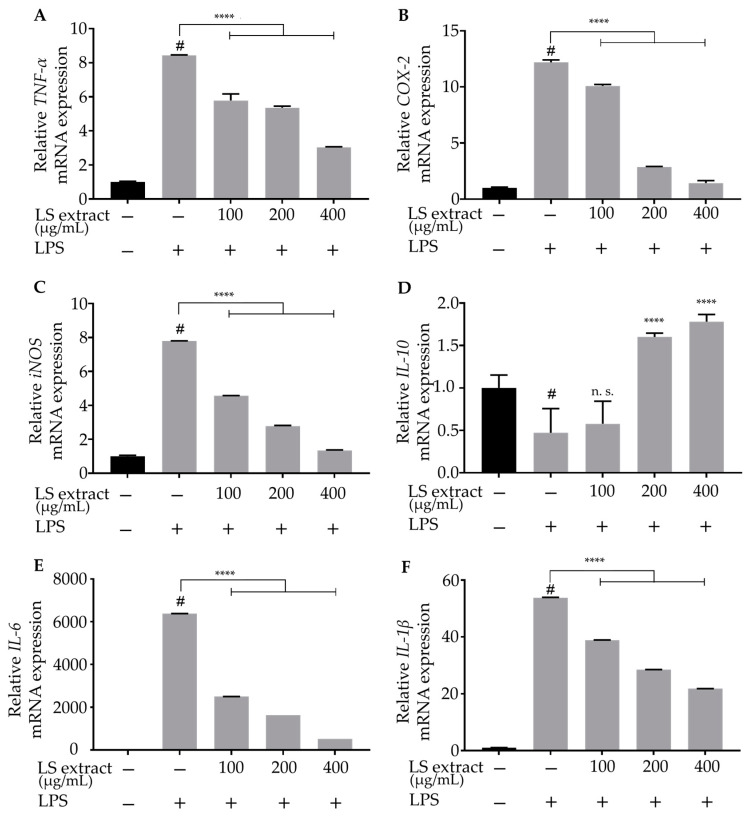
Effects of LS leaf extract on the expression of pro-inflammatory cytokines in LPS-stimulated RAW 264.7 macrophages. RAW 264.7 cells were pretreated with LS leaf extract for 24 h, followed by LPS (1 µg/mL) stimulation for 18 h. The expression of *TNF-α* (**A**), *cyclooxygenase-2* (*COX-2*) (**B**), inducible *nitric oxide synthase* (*iNOS*) (*NOS2*) (**C**), *interleukin-10* (*IL-10*) (**D**), *IL-6* (**E**), and *IL-1β* (**F**) was analyzed by qRT-PCR (*n* = 3). Values represent the mean ± SD. Statistical significance was calculated by *t*-test and one-way ANOVA. # *p* < 0.05 vs. cells treated with media only; n. s., not significant; **** *p* < 0.0001 vs. cells treated with LPS only.

We tested whether LS leaf extract suppressed the transcription of inflammatory mediators using qRT-PCR. We analyzed three concentrations, 100, 200, and 400 µg/mL, of LS extract that are non-toxic and showed clear suppressive effects on the production of inflammatory mediators as shown in Figure 3. As expected, LS leaf extract strongly suppressed the up-regulation of *iNOS* (*NOS2*), *COX-2*, and cytokine (*TNF-α, IL-1β, IL-6*) messenger RNA (mRNA) levels (Figure 4A–C,E,F). In contrast, LS leaf extract (>200 µg/mL) significantly induced the expression of *IL-10*, an anti-inflammatory cytokine that limits the host immune response (Figure 4D). These results indicate that LS leaf extract inhibits the expression of inflammatory mediators at the transcriptional level.

### 2.5. LS Leaf Extract Inhibits Phosphorylation and Translocation of NF-ĸB in LPS-Stimulated RAW 264.7 Cells

**Figure 5:**
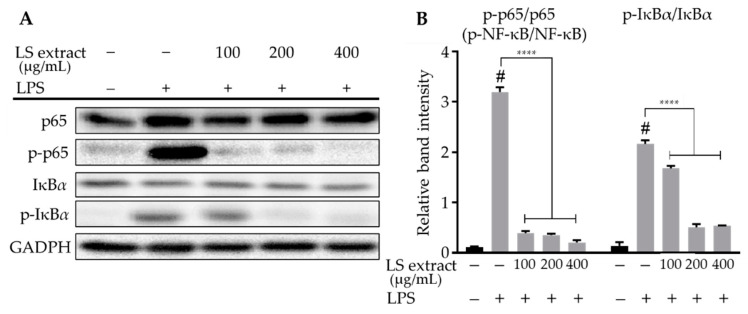
Effects of LS leaf extract on LPS-induced activation of the nuclear factor kappa B (NF-κB) pathway in RAW 264.7 cells. Cells were pretreated with LS extract for 24 h, followed by LPS (1 µg/mL) stimulation for 18 h. Whole-cell lysates were subjected to Western blotting (**A**). Glyceraldehyde 3-phosphate dehydrogenase (GAPDH) was used as a control. The relative band intensity of phosphorylated target protein to total target protein was analyzed using Image J (**B**) (*n* = 3). Values represent the mean ± SD. Statistical significance was calculated by *t*-test and one-way ANOVA. # *p* < 0.05 vs. cells treated with media only; n. s., not significant; **** *p* < 0.0001 vs. cells treated with LPS only.

NF-κB proteins are heterodimers consisting of two p50 and p65 monomers that are regulated by the degradation of nuclear factor of kappa light polypeptide gene enhancer in B-cells inhibitor, alpha (IκBα). IκBα phosphorylation induces the translocation of phosphorylated-NF-κB (p-NF-κB) from the cytoplasm to the nucleus, in order for it to transcriptionally regulate genes associated with the inflammatory processes. To investigate the effect of LS leaf extract on the regulation of the NF-κB pathway, the levels of phosphorylated p65 (p-p65) and phosphorylated-IκBα (p-IκBα) in whole-cell extracts were examined by Western blot. As shown in Figure 5, p-p65 and p-IκBα were significantly reduced by treatment with the LS leaf extract (100–400 µg/mL).

**Figure 6:**
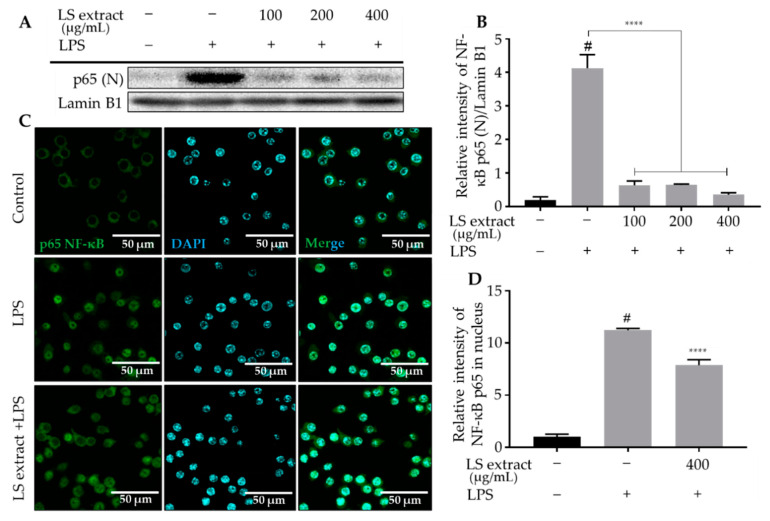
The inhibitory effects of LS leaf extract on translocation of the p65 NF-κB subunit in LPS-stimulated RAW 264.7 cells. Cells were pretreated with LS leaf extract for 24 h, followed by LPS (1 µg/mL) stimulation for 18 h. The nuclear (N) p65 NF-κB subunit was detected by Western blotting (*n* = 3) (**A**). The relative band intensity of p65 (N) to lamin B1 (nuclear loading control) was quantified using Image J (**B**). Cells were pretreated without or with 400 µg/mL of LS leaf extract followed by LPS stimulation, and then labeled with an anti-NF-κB p65 antibody tagged with Alex Fluor 488 (Green) and nuclear-counterstaining 4′,6-diamidino-2-phenylindole (DAPI; blue) (**C**). The fluorescent intensity of nuclei was analyzed using MetaMorph software (*n* = 30) (**D**). Control, cells treated with only culture medium; LPS, cells treated with LPS only (1 µg/mL); LS extract + LPS, cells treated LS leaf extract (400 µg/mL) followed by LPS (1 µg/mL). Values represent the mean ± SD; scale bar, 50 µm. Statistical significance was calculated by *t*-test and one-way ANOVA. # *p* < 0.05 vs. cells treated with media only; n. s., not significant; **** *p* < 0.0001 vs. cells treated with LPS only.

Moreover, levels of the p65 NF-κB subunit in the nucleus also significantly decreased in response to LS leaf extract treatment (100–400 µg/mL) (Figure 6A,B). The p65 NF-κB subunit was visualized using a fluorescence-labeled antibody to confirm the effect of LS leaf extract on its translocation. Consistent with the Western blot results, LS leaf extract inhibited the translocation of p65 from the cytoplasm to the nucleus (Figure 6C,D). These data indicate that the LS leaf extract controls the inflammatory response of RAW 264.7 cells by inhibiting the activation of NF-κB.

### 2.6. LS Leaf Extract Inhibits the Phosphorylation of MAPKs and Akt in LPS-Stimulated RAW 264.7 Cells

**Figure 7:**
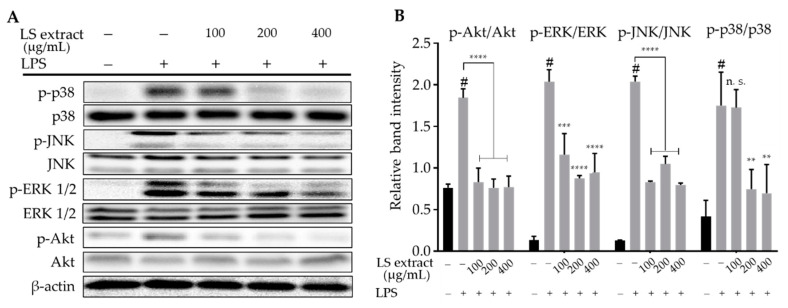
Effects of LS leaf extract on LPS-induced activation of mitogen-activated protein kinases (MAPKs) and phosphoinositide-3-kinase/protein kinase B (PI3K/Akt) pathways in RAW 264.7 cells. Cells were pretreated with LS leaf extract for 24 h, followed by LPS (1 µg/mL) stimulation for 18 h. The expression of p-p38, p38, p-c-Jun N-terminal kinase (JNK), JNK, p-extracellular signal-regulated kinases 1/2 (ERK 1/2), ERK1/2, p-Akt, and Akt was analyzed by Western blot (**A**). β-actin was used as a loading control. The relative band intensity of phosphorylated (p-) target protein compared to total target protein was quantified using Image J (**B**) (*n* = 3). Values represent the mean ± SD. Statistical significance was calculated by *t*-test and one-way ANOVA. # *p* < 0.05 vs. cells treated with media only; n. s., not significant; ** *p* < 0.01, *** *p* < 0.001, and **** *p* < 0.0001 vs. cells treated with LPS only.

The MAPKs and PI3K/Akt signaling pathways are upstream of NF-κB signaling pathways, and play a critical role in LPS-induced inflammatory responses in RAW 264.7 cells [7,21]. Our Western blot results showed that LS leaf extract effectively reduced the phosphorylation of MAPKs (p38, ERK, JNK) and Akt (Figure 7A,B). Thus, LS leaf extract suppresses the inflammatory responses by deactivating the MAPKs and PI3K/Akt signaling pathways in LPS-stimulated RAW 264.7 cells.

### 2.7. LS Leaf Extract Promotes the Activation of Nrf2/HO-1 Pathway in LPS-Stimulated RAW 264.7 Cells

**Figure 8:**
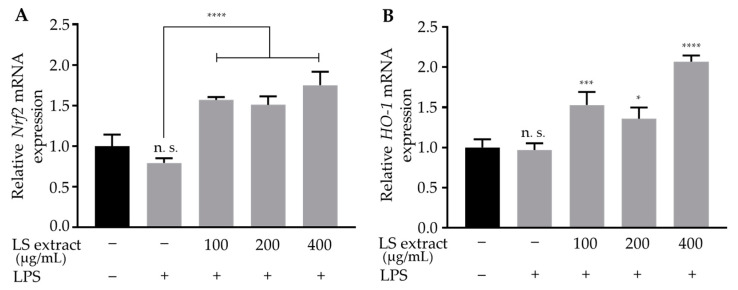
Effects of LS leaf extract on the expression of transcription factor *nuclear factor-erythroid-2-related factor* (*Nrf2*) (**A**) and phase II enzyme *heme oxygenase-1* (*HO-1*) (**B**) in LPS-stimulated RAW 264.7 cells. Cells were pretreated with LS leaf extract for 24 h, followed by LPS (1 µg/mL) stimulation for 18 h. The expression of *Nrf2* and *HO-1* genes was analyzed by qRT-PCR (*n* = 3). Values represent the mean ± SD. Statistical significance was calculated by *t*-test and one-way ANOVA. n. s., not significant; * *p* < 0.05, *** *p* < 0.001, and **** *p* < 0.0001 vs. cells treated with LPS only.

The Nrf2/HO-1 signaling pathway plays an essential role in intracellular antioxidant systems [22]. To analyze the effect of LS leaf extract on the Nrf2/OH-1 pathway, we firstly measured the mRNA levels of transcription factor *Nrf2* and *HO-1* by qRT-PCR. *Nrf2* and *HO-1* mRNA levels were significantly upregulated by pretreatment of LPS-stimulated RAW cells with LS leaf extract (100–400 µg/mL) (Figure 8A,B). Although pretreatment with 400 µg/mL of LS extract showed the highest effect, the upregulation of *Nrf2* and *HO-1* seems not to be dose-dependent with respect to LS extract. Further studies are needed to address the reason(s) behind this observation.

**Figure 9:**
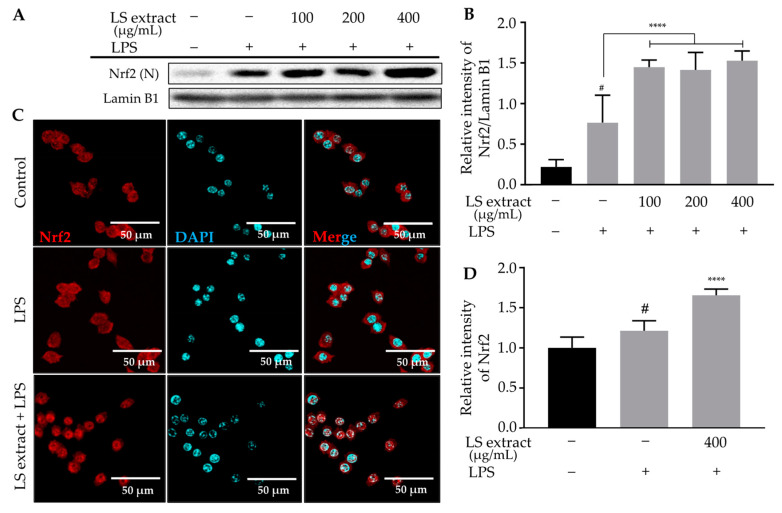
Effects of LS leaf extract on Nrf2 translocation in LPS-stimulated RAW 264.7 cells. Cells were pretreated with LS leaf extract for 24 h, followed by LPS (1 µg/mL) stimulation or 18 h. Nuclear (N) Nrf2 was analyzed by Western blot (*n* = 3) (**A**). Lamin B1 was used as a nuclear loading control. The relative band intensity of Nrf2 (N) compared to lamin B1 was quantified using Image J (**B**). The cells were treated with culture medium only (control), LPS only (1 µg/mL), or LS leaf extract (400 µg/mL) and LPS (1 µg/mL), and stained with an Nrf2 antibody labeled with Alex Fluor 594 (red) and DAPI (blue). Representative images acquired by confocal fluorescence microscopy are shown in (**C**). Fluorescent intensity was analyzed using MetaMorph software (**D**) (*n* = 30). Values represent the mean ± SD; scale bar, 50 µm. Statistical significance was calculated by *t*-test and one-way ANOVA. # *p* < 0.05 vs. cells treated with media only; n. s., not significant; **** *p* < 0.0001 vs. cells treated with LPS only.

Next, Western blotting of nuclear proteins demonstrated that LS leaf extract promoted Nrf2 nuclear translocation in RAW 264.7 cells (Figure 9A,B). Furthermore, the location of Nrf2 in the nucleus was confirmed by immunostaining (Figure 9C,D). These results imply that the anti-inflammatory properties of LS leaf extract relate to induction of the Nrf2/HO-1 signaling pathway.

## 3. Discussion

Inflammation is described as an immune response that defends against infection or injury. When our body fails to respond to inflammation, tissues are destroyed and lose their function, a phenomenon which is characteristic of many serious diseases such as diabetes, cardiovascular disorders, Alzheimer’s disease, autoimmune and pulmonary disease, and cancer. Currently, epidemiological evidence suggests that plants have various bioactivities that are associated with anti-inflammatory effects. Therefore, traditional medicines or natural products are potential sources of novel anti-inflammatory active compounds that could lead to the innovative therapeutics with fewer toxic side effects [23,24]. LS was previously reported to have many pharmacological effects that are used in traditional Vietnamese medicine to treat inflammatory diseases [12,13,25]. However, the mechanisms behind its pharmacological properties and its chemical constituents are not yet elucidated. We found that, consistent with previous studies, LS leaf extract had high DPPH and ABTS scavenging activity [13,26]. Antioxidants act as oxygen scavengers capable of catalyzing the oxidative process [27]. Lin et al. (2016) showed that polyphenolic is the major donor in the anti-oxidative process [28]. LS leaf extract possessed anti-oxidative ability, likely due to its high polyphenolic content (158 ± 0.56 mg GAE/g of dried extract, 20.08 mg ± 0.07 mg GAE/g dried leaf) compared to other plant extracts. For example, ethanol extract of the *Aniseia martinicense* leaf contains phenolics, 10.61 ± 3.12 mg GAE/g of dried leaf, which is much lower than that of the LS leaf extract [29]. Additionally, both ginger and turmeric are herbs that possess powerful anti-oxidant and anti-inflammatory properties, which could help decrease pain and protect against disease [30]. In this study, polyphenolic compounds were found to be good scavengers of free radicals to a much higher than previously reported on these herbs [31,32]. This suggests that the *L. spinosa* leaf should not only be further evaluated at a scientific level, but also as an adjunct herb that may potentially have wide applications.

Antioxidants have important roles in redox mechanisms by protecting the cell against inflammation and apoptosis [28]. Oxidative stress caused by LPS or other stimuli may trigger the activation of macrophages, thereby leading to an excessive inflammatory response [33]. As illustrated in Figure 2A and 2B, the high concentration of LS leaf extract (>400 µg/mL) reduced cell viability, suggesting that an overdose of LS leaf extract or its active components may be toxic, giving rise to cell damage. In contrast, cell viability and morphology analyses revealed that LS leaf extract (50–400 µg/mL) protects RAW 264.7 cells from LPS-induced damage (Figure 2C,D).This effect may relate to the anti-oxidative and anti-inflammatory effects of the extract. Here, we clearly showed that LS leaf extract suppressed LPS-induced production of the important inflammatory mediator TNF-α, intracellular ROS, and NO in RAW 264.7 macrophages (Figure 3). This suggests that LS leaf extract can alleviate several inflammatory symptoms as a result of its anti-inflammatory properties.

Pro-inflammation cytokines (TNF-α, IL-6, IL-1β) and enzymes (iNOS, COX-2) are induced by the immune system during inflammation. Interestingly, our extract inhibited the expression of these cytokines and enzymes (Figure 4). Noticeably, IL-6 and TNF-α are the primary mediators of local inflammation and sepsis [34]. Elevated expression of IL-1β was observed in various human cancers, such as melanoma, colon, breast, and lung cancers [35]. On the other hand, IL-10 is a potent anti-inflammatory cytokine that controls the inflammatory processes by inhibiting various pro-inflammatory cytokines [36,37]. Here, we found that the LS leaf extract enhances *IL-10* expression in LPS-stimulated macrophages (Figure 4D). Previous studies suggested that IL-10 induction may exert beneficial effects in reducing the chronic inflammatory response [38]. This suggests that the LS leaf extract not only inhibits the inflammatory response but also promotes anti-inflammatory factors. Thus, further research should aim to uncover the exact mechanism(s) behind the anti-inflammatory effects of LS leaves in order to develop drugs for inflammatory diseases.

The activation of innate immunity by LPS starts with its binding to TLR-4, a receptor for pathogen-derived molecules such as LPS and lipoteichoic acid [39]. TLR-4 activation leads to activation of the canonical NF-κB signaling pathway. At basal conditions, the NF-κB p50/p65 heterodimers are sequestered in the cytoplasm through the formation of a complex with inhibitory IκB [40]. When macrophages are stimulated by LPS, IκB is rapidly phosphorylated, leading to its degradation by the ubiquitination-mediated 26S proteasome. NF-κB dimers are subsequently liberated and translocated to the nucleus to promote the expression of pro-inflammatory cytokines [41,42]. Our study showed that LS leaf extract strongly inhibits the phosphorylation of IκBα and the NF-κB p65 subunit (Figure 5) and the nuclear translocation of NF-κB p65 in LPS-stimulated RAW 264.7 cells (Figure 6), suggesting that the activation of TLR-4 pathway is suppressed. Apart from functioning as modulators of the inflammatory response, proinflammatory cytokines also facilitate the induction of free radicals such as NO and ROS. In addition, the excessive appearance of free radicals (ROS and NO) leads to a state of oxidative stress; thus, free radicals can both activate and repress NF-κB signaling [43,44]. The reduction of intracellular ROS by LS leaf extract may, therefore, affect the activation of NF-κB signaling. In TLR-4-stimulated cells, MyD88 and TRIF are recruited to phosphorylate interleukin-1 (IL-1)-receptor-associated kinase (IRAK) and its downstream targets to activate NF-κB signaling, which is necessary to initiate the expression of pro-inflammatory cytokines [45]. Thus, we cannot exclude the possibility that LS leaf extract inhibits these molecules, thereby affecting activation of the TLR-4 pathway. In addition, we found that LS leaf extract strongly reduced pro-inflammatory factors (Figure 4), suggesting that activation of the TLR-4 signaling pathway is inhibited by LS leaf extract in the initial steps of inflammation.

NF-κB activation is related to several signaling pathways, including MAPKs and PI3K/Akt pathways that are crucial for regulating inflammation and producing inflammatory factors [46,47]. Previous studies showed that LPS stimulation increases the phosphorylation of MAPKs (JNK, p38 MAPK, ERK) in macrophages [48]. PI3K/Akt signal transduction involves the activation of the TLR-4 pathway by LPS [43]. Li et al. reported that Akt (downstream of the NF-κB, MAPKs, and interferon regulatory factor-3 (IRF-3) pathways) is indirectly involved in the activation of macrophages by *Astragalus* polysaccharide [49]. Saponaro et al. reported that LPS-induced p-Akt activation occurs upstream of NF-κB activation in microglia [50]. Despite the discrepancy in the position of TLR-4 in the pathway, these reports suggest the involvement of PI3K/Akt in the activation of NF-κB-dependent inflammatory responses. Our data (Figure 7) indicated that LS leaf extract inhibits the phosphorylation of MAPKs (p38, ERK, JNK) and Akt in response to LPS stimulation. These results demonstrate that LS leaf extract may suppress the inflammatory response by inhibiting the activation of MAPKs and PI3K/Akt. Previous reports described that the suppression of NF-κB and MAPK pathway activation leads to the inhibition of NO, PGE_2_, iNOS, COX-2, TNF-α, and IL-6 production in LPS-stimulated RAW 264.7 cells [47]. Taken together, our evidence reinforces the hypothesis that the anti-inflammatory capacity of LS leaf extract relates to the regulation of the TLR-4 pathway in LPS-stimulated RAW 264.7 cells. 

In this study, we demonstrated that LS leaf extract has strong anti-oxidant effects (Figure 1), possibly due to its high levels of polyphenolic compounds. A previous study showed that LPS causes oxidative stress that may trigger the activation of macrophages, leading to an excessive inflammatory response [51]. Therefore, we further analyzed the anti-oxidant effects underlying the inhibition of LPS-stimulated macrophage activation. LPS stimulation rapidly increases the ROS level in macrophages. This response not only causes DNA damage but also leads to overproduction of inflammatory cytokines and enzymes [52]. Nrf2 is an upstream mediator of the antioxidant response element. Under normal conditions, Nrf2 is anchored in its inactive form in the cytoplasm through its interaction with Kelch-like encoded in human (ECH)-associated protein (Keap1), a sensor of oxidative signals and electrophilic reactions. In response to oxidative stress, Nrf2 dissociates from Keap1, translocates to the nucleus, and induces the expression of phase II anti-oxidative enzymes such as HO-1, glutathione synthesis enzyme, and drug transporters. We found that pretreatment with LS leaf extract increased the expression of *Nrf2* and *HO-1* (Figure 8), repressed the expression of *iNOS* and *COX-2* (a PGE_2_-producing enzyme) (Figure 4), and suppressed the production of ROS and NO (Figure 3) in LPS-stimulated RAW 264.7 cells. Moreover, nuclear translocation of Nrf2 was enhanced by pretreatment with LS leaf extract (Figure 9). These findings illustrate that LS leaf extract is able to activate the Nrf2/HO-1 pathway to promote the downstream expression of HO-1, in order to reduce ROS production. These findings coincide with the findings from Yao et al. (2019) that nardochinoid B isolated from *Nardostachys chinensis* inhibits the activation of RAW264.7 cells stimulated by LPS through activation of the Nrf2/HO-1 pathway [53]. However, nardochinoid B did not inhibit the activation of the NF-κB and MAPK pathways. Polyphenols are considered anti-oxidative and anti-inflammatory agents due to their effects on radical scavenging, inhibition of certain enzymes involved in ROS production, and upregulation of endogenous antioxidant enzymes [54]. Therefore, activation of the Nrf2/HO-1 pathway in LPS-stimulated macrophages may be promoted by the polyphenols in LS leaf extract.

Previous studies mentioned that TLR-4 plays a crucial role in various diseases, especially cancer [55,56]. In the present study, we demonstrated that LS leaf extract inhibits the inflammatory response in LPS-stimulated macrophages by not only inhibiting TLR-4 activation but also promoting the Nrf2/HO-1 pathway. Interestingly, under the investigated conditions, pretreatment of LS leaf extract induced cell death at a high concentration. However, at a low concentration, it protected cells from the cytotoxicity effect of LPS-induced inflammation. Furthermore, our data suggest that the effect of LS leaf extract, in a concentration-dependent manner, might suppress inflammation by LPS-induced RAW 264.7 cells. Thus, *L. spinosa* leaf extract may contain components that are either beneficial or harmful to cells. To address this hypothesis, further studies are needed to identify potentially active compounds and to assess the effects of anti-inflammatory agents. In conclusion, this study provides the first evidence that LS leaf extract suppresses the inflammatory response. Therefore, LS may be a promising alternative anti-inflammatory agent.

## 4. Materials and Methods

### 4.1. Preparation of LS Leaf Ethanol Extracts

LS leaves were collected in An Giang province, Vietnam (lat. 10°22′52′’ north latitude and 105°25′12′’ east longitude), dried naturally, and ground with a waring blender. Then, 10 g of dried powder was extracted with 100 mL of ethanol (FUJIFILM-Wako, Tokyo, Japan) at 25 °C, before centrifuging to collect the supernatant, resulting in 1.27 g of extract. The residue was extracted three more times and supernatants were combined. The extract was concentrated under reduced pressure using a rotary evaporator (Rotavapor R-300, BUCHI, Switzerland), freeze-dried, and then stored at 4 °C until use. Voucher specimens of LS were deposited in the Laboratory of Plant Biology, Department of Biology, Can Tho University, Vietnam under code number (Ls01.2018-AG006).

### 4.2. Radical Scavenging Activity and Total Polyphenolic Content

The radical scavenging effects of LS leaf extract on 2,2-diphenyl-1-picrylhydrazyl (DPPH; Sigma-Aldrich, MA, USA) and 2,2-azino-bis(3-ethylbenzothiazoline-6-sulfonic acid) diammonium salt (ABTS; Sigma-Aldrich) radicals were evaluated by previously described methods with slight modifications [57,58]. Briefly, various concentrations of LS leaf extracts (0, 4, 8, 12, 16, 20, 28 µg/mL) in 0.96 mL of methanol were mixed with 0.04 mL of DPPH in methanol (1 mg/mL). The mixtures were incubated in the dark at room temperature for 30 min. The absorbance was measured at 517 nm using a V-730 UV–Vis Spectrophotometer (Jasco, Tokyo, Japan). Vitamin C (FUJIFILM-Wako) was used as a positive control.

For the ABTS assay, the stock solution was prepared by mixing 0.2 mL of 7 mM ABTS solution with 0.2 mL of 2.4 mM potassium persulfate solution (pH 7.4) and allowed to generate ABTS radicals by incubation in the dark at room temperature for 14 h. The solution was then diluted with methanol to obtain an absorbance of 0.71 ± 0.01 at 734 nm. The scavenging activity was measured by mixing 1 mL of ABTS solution with 0.25 mL of various concentrations of LS sample, and the absorbance was measured at 734 nm after 6 min of incubation. Trolox (6-hydroxy-2,5,7,8-tetramethyl chromane 2-carboxylic acid, FUJIFILM-Wako) was used as a positive control.

In both DPPH and ABTS assays, the scavenging activity was calculated with the following Equation (1):

(1) $$\mathrm{Radical}\;\mathrm{scavenging}\;\mathrm{activity}\;\left(\%\right)=\frac{\left(A_{\mathrm{cs}}-A_{\mathrm{Blank}\;1}\right)-\left(A_{s}-A_{\mathrm{Blank}\;2}\right)}{A_{\mathrm{cs}}-A_{\mathrm{Blank}\;1}}$$

where Acs is the absorbance of the control reaction (without test or standard sample), As is the absorbance in the presence of test or standard sample, A_Blank 1_ is the absorbance of Blank 1 containing only methanol, and A_Blank 2_ is the absorbance of Blank 2 containing test or standard sample in methanol.

The total polyphenolic content of LS leaf extract was determined using the Folin–Ciocalteu reagent with a slight modification [59]. A volume of 0.25 mL of plant extract in methanol (50 µg/mL) was mixed with 0.25 mL of the Folin–Ciocalteu reagent and neutralized with 0.25 mL of 10% sodium carbonate solution (*w*/*v*). The reaction mixture was incubated at 40 °C for 30 min with intermittent shaking for color development and then the absorbance at 765 nm was measured. Gallic acid (FUJIFILM-Wako), was used as a control for generating the calibration curve. The total polyphenolic content was expressed as mg/g gallic acid equivalent (GAE) of dried leaf.

### 4.3. Cell Culture, Cell Viability, and LDH Activity

RAW 264.7 murine leukemia macrophage cells (ATCC TIB-71^TM^, Manasas, VA, USA) were maintained with Dulbecco’s modified Eagle’s medium (DMEM; FUJIFILM-Wako, Tokyo, Japan) containing 10% fetal bovine serum (FBS; GIBCO BRL, Grand Island, NY, USA) and 1% penicillin–streptomycin (PS; FUJIFILM-Wako, Tokyo, Japan) in a humidified incubator containing 5% CO_2_ at 37 °C.

For cell experiments, LS leaf ethanol extract (1 g/mL) was dissolved in 5% (*v*/*v*) dimethyl sulfoxide (DMSO; FUJIFILM-Wako, Tokyo, Japan) as a stock. The stock solution was diluted to an appropriate sample concentration with DMEM and then added to the culture medium in 96-well plates that were incubated for 24 h. During treatment, the DMSO concentration was kept at 0.5% (*v*/*v*).

Cell viability was measured using the Cell Counting Kit-8 assay (CCK-8; Dojindo Molecular Technologies Inc., Rockville, MD, USA), which is based on the measurement of intracellular dehydrogenase activity. Briefly, the cells were treated with serial concentrations of LS leaf extract (0, 50, 100, 200, 400, 800, and 1000 µg/mL) for 24 h in 96 well-plates at a density of 2 × 10^4^ cells/well, followed by incubation with or without 1 µg/mL LPS (from *Escherichia coli*, Sigma-Aldrich, St. Louis, MO, USA) for 18 h. Then, absorption at 450 nm was read using an SH-1200 microplate reader (CORONA electric, Ibaraki, Japan). To assess cell damage, LDH activity in the medium was measured using the Cytotoxicity LDH assay Kit-WST (Dojindo Molecular Technologies, Tokyo, Japan). Whole-cell extracts were prepared by cell lysis using 2% (*v*/*v*) Triton X-100.

### 4.4. NO Production and ROS Accumulation

RAW 264.7 cells were pretreated with the indicated concentration of LS extract dissolved in DMEM for 24 h, the medium was removed, and cells were washed with phosphate-buffered saline (PBS). Then, cells were stimulated with 1 µg/mL LPS in DMEM for 18 h. To quantify NO, the conditioned medium (0.1 mL) was removed and mixed with an equal volume of Griess reagent (FUJIFILM-Wako) dissolved in water for 10 min at room temperature. The absorbance at 540 nm was measured with an SH-1200 microplate reader, and NO concentration was calculated based on a standard curve generated with sodium nitrite [60]. *N*-Nitro-***L***-arginine methyl ester (L-NAME; FUJIFILM-Wako) (100 μM) was used as a standard iNOS inhibitor (positive control).

ROS production was assayed using the Cellular ROS assay Kit (Abcam, Tokyo, Japan). After removing the media, the cells were stained with 10 µM 2’,7’-dichlorodihydrofluorescein diacetate (DCFH_2_ –DA) at 37 for 45 min. The fluorescence, which corresponds to the intracellular ROS level, was measured using a Wallac 1420 spectrofluorometer (Perkin-Elmer, Turku, Finland) at an excitation wavelength of 488 nm and an emission wavelength of 535 nm.

### 4.5. ELISA

The expression level of TNF-α protein in LPS-stimulated RAW 264.7 cells was analyzed by ELISA using the TNF-α ELISA Ready-SET-Go Kit (eBiosciences, San Diego, CA, USA). TNF-α concentration was calculated based on the standard curve made using the TNF-α mouse standard set in the kit. RAW 264.7 macrophages were incubated with 1 µg/mL LPS in the presence of various concentrations of LS extract for 24 h. The supernatants were collected and stored at −80 °C until analysis.

### 4.6. Quantitative RT-PCR (qRT-PCR)

RAW 264.7 cells were incubated for 24 h in six-well plates with LS leaf extract, washed with PBS, and then stimulated with or without LPS 1 µg/mL for 18 h. Total RNA was extracted using Trizol reagent (Invitrogen; Carlsberg, CA, USA) followed by purification with the Qiagen RNeasy Kit (Qiagen; Hilden, Germany). Complementary DNA (cDNA) was synthesized using Transcriptor Universal cDNA Master Mix (Roche, Mannheim, Germany) and a SimpliAmp™ Thermal Cycler (Life Technologies, Singapore) according to the manufacturer’s instructions. Quantitative polymerase chain reaction (PCR) was performed using the FastStart Essential DNA Green Master Mix (Roche, Mannheim, Germany) and a Light-Cycler 96 (Roche). *β-Actin* was used as a normalization control. The primers used in this study are listed in Table 1.

### 4.7. Western Blotting

RAW 264.7 cells were seeded in six-well plates and incubated with different concentrations of LS leaf extract for 24 h prior to stimulation with 1 µg/mL LPS for 18 h. Total protein was extracted from cells using Radioimmunoprecipitation assay (RIPA) lysis buffer (FUJIFILM-Wako) containing a cocktail of protease and phosphatase inhibitors. Cytoplasmic and nuclear proteins were separated using a Nuclear Extraction Kit (Abcam), and protein concentrations were analyzed using a Pierce ^TM^ BCA protein assay kit (Thermo Fisher Scientific, Waltham, MA, USA). The protein samples were separated by 10 % SDS-PAGE and transferred onto polyvinylidene fluoride (PDVF) membranes. After blocking for 1 h with a solution containing 5% skim milk (FUJIFILM-Wako), the membrane was incubated with the primary antibodies overnight at 4 ℃. The primary antibodies and phospho-specific antibodies (IκB*α*, p-IĸB*α*, p-NF-κB p65, p38, p-p38, ERK1/2, p-ERK1/2, JNK, Akt, p-Akt, β-actin (Cell Signaling Technology, Tokyo, Japan), p-JNK, NF-κB p65 (Sigma-Aldrich), Nrf2 (Proteintech, Japan), Lamin-B1, and GAPDH (Santa Cruz Biotechnology, Dallas, TX, USA)) were diluted according to the manufacturer’s recommendation. After washing in Tris-buffered saline containing 0.5% Tween-20 (TBST), the membrane was incubated with horseradish-peroxidase-conjugated secondary antibody (Sigma-Aldrich) in TBST containing 5% skim milk or 3% bovine serum albumin (BSA fraction V; Roche, Mannheim, Germany) at room temperature for 1 h. Proteins were visualized using Pierce^TM^ Enhanced chemiluminescent (ECL) Western Blotting Substrate (Thermo Fisher Scientific). Quantification of band intensity was performed using Image J software (National Institute of Health, Sacaton, Arizona, AZ, USA) and normalized to β-Actin or GAPDH.

### 4.8. Immunofluorescent Staining

Cells were seeded in eight-well chamber slides (Thermo Fisher Scientific, Nunc™ Lab-Tek^®^ Chamber Slides, Waltham, MA, USA). After treatment with LS leaf extract followed by LPS stimulation, the cells were fixed with 4% paraformaldehyde phosphate buffer solution (FUJIFILM-Wako) according to previously described methods [61]. Cells were incubated with rabbit anti-NF-κB p65 (1:500) followed by anti-rabbit immunoglobulin G (IgG) Alexa Fluor^TM^ 488-conjugate (1:800; Molecular Probes, Invitrogen, Carlsbad, CA, USA), or rabbit anti-Nrf2 (1:500) followed by anti-rabbit IgG Alexa Fluor^TM^ 594-conjugate (1:800; Molecular Probes). For staining of nuclei, 4′,6-diamidino-2-phenylindole (DAPI; Molecular Probes, Eugene, OR, USA) was used for 30 min. Samples were then mounted on a glass slide in Vectashield mounting medium (Vector Laboratories, Tokyo, Japan) and inspected using a fluorescence FV10i microscope (Olympus, Tokyo, Japan). The fluorescent intensity was analyzed using MetaMorph software (version 7.7.7.0; Molecular Devices, Sunnyvale, CA, USA).

### 4.9. Statistical Analysis

Experiments were repeated at least three times, and all data are expressed as means ± SD. Statistical significance was evaluated using *t*-tests and one-way ANOVAs, where *p* < 0.05 was considered significant.

## Figures and Tables

**Table 1:** Primers used for qRT-PCR analysis.

Gene ^1^	Primer Sequence	
	Forward Primer (5′–3′)	Reverse Primer (5′–3′)
*iNOS (NOS2)*	GGAGCCTTTAGACCTCAACAGA	AAGGTGAGCTGAACGAGGAG
*IL-6*	GCTACCAAACTGGATATAATCAGGA	CCAGGTAGCTATGGTACTCCAGAA
*IL-1* *β*	AGTTGACGGACCCCAAAAG	AGCTGGATGCTCTCATCAGG
*TNF-α*	CTGTAGCCCACGTCGTAGC	TTGAGATCCATGCCGTTG
*IL-10*	CGCTTGGAATCCCGAATTA	CTCAGGTTGGTCACAGTGAAAT
*COX-2*	GATGCTCTTCCGAGCTGTG	GGATTGGAACAGCAAGGATTT
*HO-1*	AGGGTCAGGTGTCCAGAGAA	CTTCCAGGGCCGTGTAGATA
*Nrf2*	CATGATGGACTTGGAGTTGC	CCTCCAAAGGATGTCAATCAA
*β-Actin*	GGAGGGGGTTGAGGTGTT	GTGTGCACTTTTATTGGTCTCAA

^1^ Primer’s name; nitric oxide synthase-2 (NOS2), interleukin-6 (IL-6), interleukin-1β (IL-1β), tumor necrosis factor-α (TNF-α), interleukin-10 (IL-10), cyclooxygenase-2 (COX-2), heme oxygenase-1 (HO-1), nuclear factor erythroid-2-related factor 2 (Nrf2), β-Actin.

## Abbreviations

LS	Lasia spinosa
DMSO	Dimethyl sulfoxide
ELISA	Enzyme-linked immunosorbent assay
LPS	Lipopolysaccharide
MAPK	Mitogen-activated protein kinase
NF-κB	Nuclear factor-kappa B
PI3K/Akt	Phosphoinositide-3-kinase–protein kinase B/Akt
HO-1	Heme oxygenase-1
L-NAME	*N*-Nitro-l-arginine methyl ester
Nrf2	Nuclear factor erythroid-2-related factor 2
ROS	Reactive oxygen species
NO	Nitric oxide
TLR-4	Toll-like receptor 4
TRIF	TIR-domain-containing adapter-inducing interferon-β
IRAK	Interleukin-1 (IL-1)-receptor-associated kinase
IRF-3	Interferon regulatory factor-3
PGE_2_	Prostaglandin E2
